# Antioxidant Therapy in Oxidative Stress-Induced Neurodegenerative Diseases: Role of Nanoparticle-Based Drug Delivery Systems in Clinical Translation

**DOI:** 10.3390/antiox11020408

**Published:** 2022-02-17

**Authors:** Anushruti Ashok, Syed Suhail Andrabi, Saffar Mansoor, Youzhi Kuang, Brian K. Kwon, Vinod Labhasetwar

**Affiliations:** 1 Biomedical Engineering, Lerner Research Institute, Cleveland Clinic, Cleveland, OH 44195, USA; ASHOKA2@ccf.org (A.A.); andrabs@ccf.org (S.S.A.); mansoos2@ccf.org (S.M.); kuangy@ccf.org (Y.K.); 2 Department of Orthopaedics, Faculty of Medicine, University of British Columbia, Vancouver, BC V5Z 1M9, Canada; brian.kwon@ubc.ca

**Keywords:** neurodegeneration, reactive oxygen species, inflammation, polymers, CNS, antioxidant enzymes

## Abstract

Free radicals are formed as a part of normal metabolic activities but are neutralized by the endogenous antioxidants present in cells/tissue, thus maintaining the redox balance. This redox balance is disrupted in certain neuropathophysiological conditions, causing oxidative stress, which is implicated in several progressive neurodegenerative diseases. Following neuronal injury, secondary injury progression is also caused by excessive production of free radicals. Highly reactive free radicals, mainly the reactive oxygen species (ROS) and reactive nitrogen species (RNS), damage the cell membrane, proteins, and DNA, which triggers a self-propagating inflammatory cascade of degenerative events. Dysfunctional mitochondria under oxidative stress conditions are considered a key mediator in progressive neurodegeneration. Exogenous delivery of antioxidants holds promise to alleviate oxidative stress to regain the redox balance. In this regard, natural and synthetic antioxidants have been evaluated. Despite promising results in preclinical studies, clinical translation of antioxidants as a therapy to treat neurodegenerative diseases remains elusive. The issues could be their low bioavailability, instability, limited transport to the target tissue, and/or poor antioxidant capacity, requiring repeated and high dosing, which cannot be administered to humans because of dose-limiting toxicity. Our laboratory is investigating nanoparticle-mediated delivery of antioxidant enzymes to address some of the above issues. Apart from being endogenous, the main advantage of antioxidant enzymes is their catalytic mechanism of action; hence, they are significantly more effective at lower doses in detoxifying the deleterious effects of free radicals than nonenzymatic antioxidants. This review provides a comprehensive analysis of the potential of antioxidant therapy, challenges in their clinical translation, and the role nanoparticles/drug delivery systems could play in addressing these challenges.

## 1. Introduction

Free radicals are generated during pivotal biological processes such as metabolic reactions, cell signaling, and gene transcription [1]. Cellular organelles such as mitochondria, peroxisomes, lysosomes, microsomes, endoplasmic reticulum, plasma membrane, and phagocytic cells are also the source of free radical production [2,3]. External factors such as environmental pollutants, radiation, smoking, heavy-metal exposure, diet, and physical exercise also contribute to the production of free radicals [4,5]. Under normal conditions, the innate antioxidative defense system that includes various enzymatic and nonenzymatic antioxidants neutralize free radicals, thus maintaining the redox balance [6]. This balance is disrupted under certain pathological conditions such as genetic mutations, inflammation, injury, ischemia/reperfusion, etc. [7,8,9]. Excessive free radicals formed overwhelm the endogenous antioxidant defense mechanism, thus causing oxidative stress which downregulates the endogenous defense system [10,11]. Neuronal cells are particularly susceptible to damage due to free radicals, as they contain high levels of unsaturated lipids that are susceptible to oxidation and the presence of high levels of redox-active transition metals that catalyze the formation of free radicals [12]. The central nervous system (CNS) has high metabolic activity and, hence, a high oxygen demand, which favors free radical formation [13]. Metabolism of neurotransmitters also produces free radicals [14]. The CNS also has a relatively weaker antioxidant defense than other organs (e.g., liver) which makes it more susceptible to oxidative stress than other organs [15,16]. Under oxidative stress condition, dysfunctional mitochondria are unable to meet the high energy need of neuronal cells for their normal biochemical and physiological functions; hence they become vulnerable to rapid cell death [17].

Pro-oxidants or free radicals are usually those atoms or molecules that contain an unpaired electron in their outermost orbit and can be formed when oxygen interacts with certain molecules [18]. These free radicals are very unstable but highly reactive and, when they interact with other molecules, create additional free radicals, initiating a self-propagating chain reaction of free radical formation [18]. Free radicals contain reactive oxygen species (ROS) and reactive nitrogen species (RNS). ROS are chemically reactive molecules containing oxygen, whereas RNS includes nitrogen (N) and oxygen (O) atoms. The ROS and RNS produced in cells comprise both free radical and non-free radical species and include hydrogen peroxide (H_2_O_2_), nitric oxide (^•^NO), nitrogen dioxide (^•^NO_2_), hydroxyl radical (^•^OH), superoxide anion (O_2_^•−^), peroxynitrite (OONO^−^), hypochlorous acid (HClO), etc. The ^•^OH radical, produced from H_2_O_2_ in the metal-catalyzed (free Fe and Cu) redox reactions such as Fenton reaction, is particularly unstable and reacts rapidly and nonspecifically with most biological molecules [3].

### 1.1. Endogenous and Exogenous Sources of Free Radicals

There are multiple cellular processes and biochemical reactions that produce free radicals as a part of normal cellular function. For e.g., during Electron Transport Chain (ETC) and its five integrated mitochondrial complexes (I, II, III, IV, and V), reduction of O_2_ to H_2_O by cytochrome c oxidase prematurely generates ROS such as singlet oxygen (^1^O_2_), O_2_^•^^−^, ^•^OH, and H_2_O_2_ [19,20,21]. Intracellular organelle, peroxisomes, responsible for degradation of fatty acids, generate H_2_O_2_ as a byproduct [22]. Neutrophils that contain myeloperoxidase (MPO) uses H_2_O_2_ and halides (Cl^−^, Br^−^, and I^−^) or pseudohalide (SCN^−^) ions to catalyze the production of free radicals [23]. Phagocytic cells (neutrophils, macrophages, and monocytes) while defending the CNS against invading microorganisms or clearing the dead cell debris produces ROS [24]. Cytochrome P450 is another intracellular enzyme present in microsomes and the endoplasmic reticulum catalyzes the ROS formation [25]. Cytosolic enzymes such as xanthine oxidase (XO) during the catalytic oxidation of hypoxanthine to xanthine and Prostaglandin H Synthase (PHS) from arachidonic acid to prostaglandin generate ROS [24]. In addition, environmental pollutants; ionizing radiation (UV-rays, X-rays, γ-rays, and infrared or electromagnetic waves); smoking; long-term chemical exposure like pesticides, insecticides, or industrial solvents; heavy or transition metals (Cu, Fe, Mn, As, Cd, Pb, and Hg); diet; and physical exercise contribute to the production of ROS/RNS [26,27,28,29,30,31,32,33,34,35,36,37,38,39].

### 1.2. Free Radicals: A Double Edge Sword

Under normal physiological conditions, low levels of ROS are essential for the regulation of critical signaling pathways involved in cell growth, proliferation, differentiation, survival, regulation of blood pressure, cognitive function, immunity, and maintaining normal antioxidant defense mechanisms of the body [40]. RNS in the CNS regulate cerebral blood flow and memory and plays a significant role in maintaining the immune system and cytokine production [41]. However, excess ROS and RNS, which are the byproducts of the oxygen and nitrogen-rich tissue environment in the body, if not neutralized by the endogenous antioxidants, results in oxidative/nitrosative stress [42]. Such conditions can damage cells by starting a chemical chain reaction and modifying biomolecules, i.e., lipids, proteins, and DNA [43]. The ROS produced by mitochondria can accelerate the oxidation of polyunsaturated fatty acids in the cell membrane lipids, a process known as lipid peroxidation (LPO) that changes the cell membrane structure, impairing its integrity, thus affecting cell signaling. The LPO products such as F2-isoprostanes, malondialdehyde (MDA), 4-hydroxynonenal (4-HNE), and oxidized low-density lipoproteins (LDL) can further damage proteins and nucleic acid bases [44]. With oxidative stress, multiple changes can occur such as mitochondrial DNA mutation, impairment in the mitochondrial respiratory chain, and change in membrane permeability influencing Ca^2+^ homeostasis [20,45,46,47].

## 2. Oxidative Stress and Neurodegenerative Diseases

**Figure 1:**
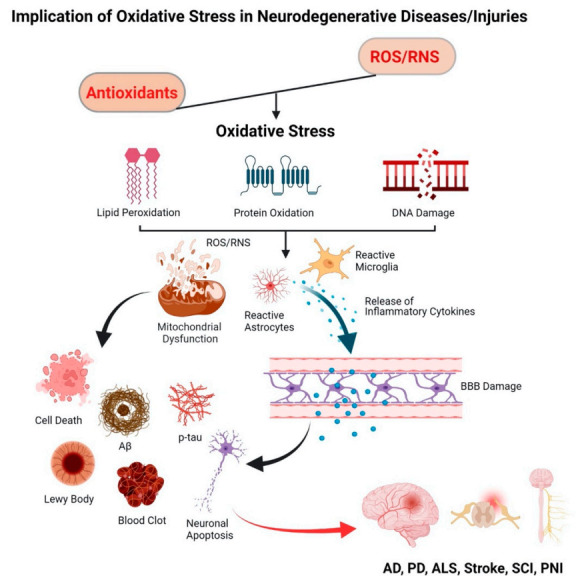
Schematic representing the effect of oxidative stress in neurodegenerative diseases. Imbalance in the level of ROS/RNS and antioxidants leads to an oxidative stress condition that causes damage to cellular biomolecules, i.e., lipids, proteins, and DNA. Mitochondrial dysfunction and accumulation of activated astrocytes and microglia release inflammatory cytokines and chemokines, promoting cellular apoptosis and tissue death.

Cell damage triggers a cascade of degenerative events via mitochondrial dysfunction, neuroinflammation, apoptosis, and tissue necrosis [20,48,49]. Oxidative stress-induced homeostatic dysregulation remains a central component of several neurodegenerative diseases such as Alzheimer’s disease (AD), Parkinson’s disease (PD), and Amyotrophic Lateral Sclerosis (ALS) [7]. Examples of injury-triggered neurodegenerative diseases include stroke, spinal cord injury (SCI), peripheral nerve injury (PNI), etc. [8,9]. The common link between these neurodegeneration conditions is oxidative stress, ineffective antioxidant defense, and mitochondrial dysfunction (Figure 1).

### 2.1. Progressive Neurodegenerative Diseases

#### 2.1.1. Alzheimer’s Disease (AD)

AD, a leading cause of dementia, is characterized by a progressive decline in cognitive function [48]. Amyloid beta (Aβ) plaques, neurofibrillary tangles (NFTs), hyperphosphorylated microtubule-associated protein tau, and neuronal loss within the brain are specific histopathological hallmarks of the AD [49]. Prior to the development of plaque pathology, oxidative stress has been recognized as the key player in the etiology of AD, contributing to mitochondrial dysfunction in synapses and neurons, and in Aβ production [50,51]. In fact, the concept of oxidative stress in AD was originally derived from the “free radical theory of aging”, meaning that free radicals play a central role in the aging process [52]. Mitochondrial dysfunction in AD includes impaired mitochondrial complexes [53,54,55,56], malfunctioning of F1Fo adenosine triphosphate (ATP) synthase, which is involved in oxidative phosphorylation [57,58], and damage to the promoter of the mitochondrial ATP synthase gene that controls ATP generation [59,60]. Further, dysfunctional mitochondria produce 4-HNE that upregulates γ-secretase complex and promotes cleavage of the amyloid precursor protein (APP), leading to Aβ accumulation [61,62]. In addition, increased Ca^2+^ and ROS levels lead to a buildup of p-tau aggregates which are toxic and are considered as one of the defining pathological hallmarks of the AD [63]. ROS also play a pivotal role in the stress kinases like the phospho-c-Jun N-terminal kinase 1 (p-JNK) pathway which is linked to tau hyperphosphorylation and cell death in response to Aβ accumulation [64]. Further, oxidative stress reduces the activities of antioxidants, i.e., superoxide dismutase (SOD), catalase (CAT), and glutathione S-transferase (GST), thus weakening the endogenous antioxidant defense of the CNS [65]. The increased levels of LPO under oxidative stress are strongly associated with neurotoxicity in AD [50] as it leads to an increase in amyloidogenesis through upregulation of β-secretase expression [66]. Although there are several downstream degenerative events, it appears that mitochondrial dysfunction and oxidative stress are the key triggering factors in the pathogenesis of AD.

#### 2.1.2. Parkinson’s Disease (PD)

PD is the second-most common neurodegenerative disease after AD that causes both motor and nonmotor symptoms [67]. The pathology of PD is driven by the accumulation and aggregation of α-synuclein, a presynaptic neuronal protein in the nervous system [68]. The mechanisms associated with the pathogenesis of PD include aberrant protein homeostasis, bioenergetic impairment, and oxidative stress [69]. Oxidative stress is associated with α-synuclein protein aggregation [64]. The cascade of events leading to degeneration of dopaminergic neurons in PD is also linked to oxidative stress [70]. Analysis of the postmortem brain tissue of the victims of PD shows elevated levels of oxidative stress markers such as 4-HNE, protein carbonyl, 8-hydroxy-2′-deoxyguanosine, and 8-hydroxy-guanosine [71]. In addition, oxidative stress is associated with the formation of Lewy bodies, which are the clumps of protein in the PD brain [72]. Experimental evidence in PD models suggests that oxidative stress in the dopaminergic neurons activates p38 mitogen-activated protein kinase (p38 MAPK) pathway that ultimately leads to apoptosis of the brain cells [73].

#### 2.1.3. Amyotrophic Lateral Sclerosis (ALS)

ALS is also known as Lou Gehrig’s disease, in which motor neurons in the brain, brain stem, and spinal cord are damaged, resulting in muscle weakness, atrophy, paralysis, and premature death [74]. Oxidative stress, mitochondrial dysfunction, and mutations in the genes that act on mitochondrial processes are involved in the pathophysiology of the ALS [75,76]. Most of the familial ALS patients (15–20%) have mutations in the superoxide dismutase 1 (SOD1) gene, which plays an important role in the defense mechanism against oxidative stress [77]. More than 150 ALS-related SOD1 gene mutations have been discovered in various parts of the enzyme, which result in protein misfolding and aggregation, increased ROS production, and redox system disequilibrium, ultimately resulting in nerve cell loss [77,78]. ALS is also linked to several interrelated risk factors, such as neuroinflammation, excitotoxicity, mitochondrial dysfunction/dysregulation, and endoplasmic reticulum stress [79,80,81]. Considerably high oxidative stress biomarkers such as MDA, 8-hydroxyguanosine, and advanced oxidation protein products are found in ALS patients [82]. In sporadic ALS patients, cystine/glutamate antiporter overexpression was observed that causes increased oxidative stress and extracellular glutamate accumulation [83]. In addition, dysregulation of the retinoic acid (RA) signaling pathway, a product of vitamin A, contributes to the death of motor neurons [84].

### 2.2. Injury-Induced Oxidative Stress

Neuronal tissue injury, physical or due to ischemic condition, is known to induce oxidative stress that triggers progressive degeneration, known as secondary injury.

#### 2.2.1. Stroke

**Figure 2:**
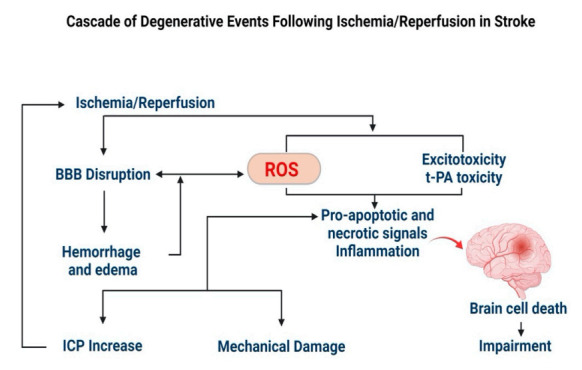
ROS-mediated degenerative events during a stroke. Excessive production of ROS during I/R injury leads to mechanical damage to the brain due to breakdown of the BBB, hemorrhage and edema, causing a build-up of intracranial pressure (ICP). The biochemical changes lead to inflammation and progression of apoptosis. Therefore, excess ROS formed during I/R is considered a target to inhibit the progression of secondary brain damage.

In stroke, thrombus formation in cerebral blood vessels creates an ischemic condition, triggering free radical formation and tissue damage (Figure 2) [85]. Resumption of blood supply to the ischemic region further exuberates the condition as more free radicals are formed, termed “reperfusion injury or reoxygenation injury” [86]. Collectively, it is referred to as the ischemia/reperfusion (I/R) injury [86]. Oxidative stress leads to mitochondrial dysfunction, neuroinflammation, and glutamate excitotoxicity, resulting in the blood-brain barrier (BBB) damage, apoptosis/necrosis of neurons, and supporting cellular elements (glial cells and vessels) [87,88,89]. These are the prominent features of neurodegeneration in the stroke-related cerebral pathology [90,91,92,93]. Further, excessive ROS production or impaired ROS degradation [94,95] stimulates vasoconstriction, increased platelet aggregation, and endothelial cell permeability, thereby affecting cerebral blood circulation [96]. Activation of matrix metalloproteinases (MMPs) disrupts the cerebral extracellular matrix (ECM), which causes immunocyte infiltration and neuroinflammation, culminating in the breakdown of the neurovascular unit (NVU), leading to hemorrhage and edema [97,98].

#### 2.2.2. Spinal Cord Injury (SCI)

**Figure 3:**
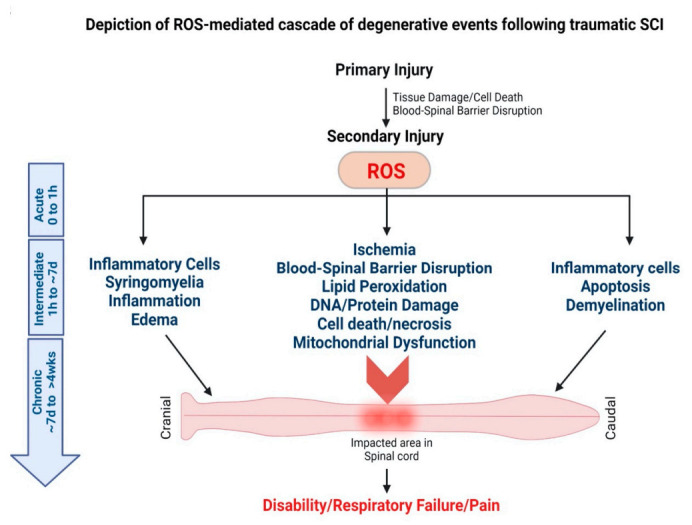
Secondary injury cascade following spinal cord injury. Traumatic injury to the spinal cord leads to secondary injury progression that affects the lesion site and the entire spinal cord, including the cranial and caudal segments of the spinal cord. Following injury, excessive production of ROS is considered to trigger the secondary injury cascade of progressive degeneration that affects the entire spinal cord.

SCI is another common form of neuronal injury that causes neurological dysfunctions [99] and is characterized by an initial primary injury followed by the secondary phase of injury (Figure 3) [100]. Primary injury results immediately from the initial trauma causing damage to the blood vessels and axons [101]. In contrast, secondary injury is the indirect result of the primary injury that involves inflammation and oxidative stress [10]. The secondary injury progression occurs not only at the site of impact, but it spreads along the entire spinal cord, including the faraway segments of the spinal cord that are not impacted, making the condition more devastating and debilitating with time [101]. Following injury, the elevated levels of ROS and the consequent oxidative stress are considered critical events associated with the secondary injury progression [102]. Under oxidative stress condition, dysfunctional mitochondria become the source of ROS [103] that cause a further cascade of degenerative processes, particularly curtailing ATP production required for normal cellular functioning, thus promoting apoptosis [103]. The excess ROS alters cell functions by modulating ion channels, followed by excessive accumulation of intracellular calcium ions that eventually causes excitotoxicity [104]. Oxidative stress damages the microvascular endothelium that reduces the spinal cord white matter blood flow, resulting in ischemic injury [105].

#### 2.2.3. Peripheral Nerve Injury (PNI)

The peripheral nervous system (PNS) is a bundle of long nerve fibers that connect different parts of the body with the CNS. Damage to the peripheral nerves due to trauma and compression can cause impairment in the brain’s communication with the target organs [106]. These injuries affect motor and sensory behaviors, perception, consciousness, and sensations of the skin and joints [106]. The most common symptoms of PNI are the defects in sensory and motor function that can lead to complete paralysis of the affected limb or the development of an intractable neuropathic pain [107]. Many surgical procedures, such as oral and maxillofacial surgery, can also cause injury to the peripheral nerves [108]. The major component of the mechanism and pathogenies of PNI involves oxidative stress and inflammation that exacerbates neural damages and plays a negative role in the regeneration process [109]. Experimental evidence at the preclinical level has demonstrated that inhibiting oxidative stress could help improve functional recovery by accelerating the repair processes [110,111,112,113,114].

Other neurodegenerative diseases implicated due to oxidative stress are: vascular dementia [115], Down syndrome [116], Autism [117], attention-deficit/hyperactivity disorder (ADHD) [118], Huntington’s disease (HD) [119], multiple sclerosis (MS) [120], depression [121], and epilepsy [122]. Similarly, in traumatic brain injury (TBI) [123], progressive degeneration occurs due to the accumulation of excessive free radicals, glutamate release, Ca^2+^ overload, mitochondrial dysfunction, leading to apoptosis/necrosis [123].

## 3. Antioxidants

From the above review of the etiology of different neurodegenerative diseases, oxidative stress is considered as the key component, whether these are chronic neurodegenerative conditions such as AD, PD, or ALS or caused by neuronal tissue injury, such as in stroke, SCI, or PNI. Dysfunctional mitochondria under oxidative stress become the main source of free radical formation and deplete the energy needed for normal cellular function, leading to inflammation and cell death [124]. Another set of literature data indicates that dysfunctional mitochondria cause oxidative stress [125]. Thus, there is a complexity in understanding the root cause, whether oxidative stress leads to mitochondrial dysfunction, or it is mitochondrial dysfunction that leads to oxidative stress [49,126,127]. Despite ambiguity on the root cause of oxidative stress, it is hypothesized that an effective treatment based on antioxidants can alleviate oxidative stress and regain the redox balance that can attenuate mitochondrial dysfunction and curtail the downstream cascade of degeneration [126]. It is also contemplated that oxidative stress-free condition can promote regeneration and healing by the endogenous mechanisms, such as by promoting migration and differentiation of progenitor and stem cells [127]. In addition, an oxidative stress-free environment could promote differentiation of macrophages preferentially to M2 phenotype, which contains growth factors and can promote healing, rather than to M1 phenotype, which contains degenerative inflammatory cytokines [128]. With this in consideration, natural and synthetic antioxidants have been evaluated in preclinical model studies and clinical trials [129].

**Figure 4:**
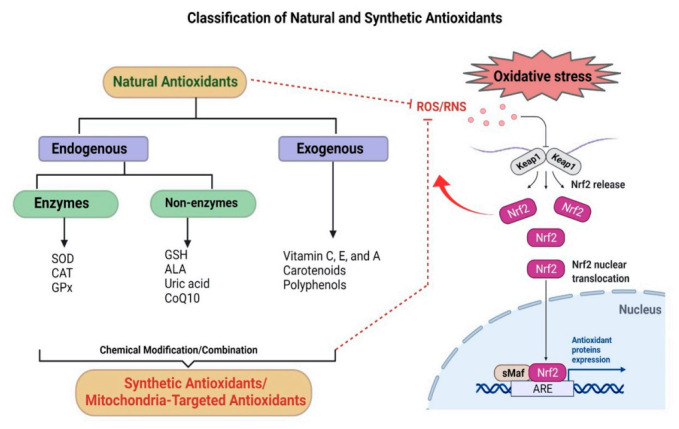
Natural and synthetic antioxidants: Classification of natural and synthetic antioxidants and the endogenous Nrf2 pathway, which regulates the activation of ARE genes. Kelch-like ECH-associated protein 1 (Keap1) represents a negative regulator of Nrf2. Under physiological conditions, Keap1 forms a ubiquitin E3 ligase complex with Cullin3 in the cytoplasm that targets Nrf2 for polyubiquitination and rapid proteasomal degradation. During oxidative stress, cysteines in Keap1 are modified and inactivated, and Nrf2 can quickly translocate into the nucleus, where it binds to small musculoaponeurotic fibrosarcoma oncogene homolog (sMaf) proteins, upregulates downstream ARE genes, and maintains redox homeostasis.

Antioxidants can reduce oxidative stress by quenching/scavenging free radical intermediates, thereby preventing oxidative chain reactions from propagating [4]. These antioxidants predominantly include various endogenous antioxidant enzymes with their substrates or coenzymes and nonenzymatic antioxidants, along with exogenous (natural and synthetic) antioxidant sources that maintain the redox equilibrium in the biological system [130]. Endogenous antioxidant activity is directly regulated by nuclear factor erythroid 2-related factor 2 (Nrf2). It is a ubiquitous redox-sensitive transcription factor that stimulates the expression of antioxidant response element (ARE)-containing gene promoters involved in ROS detoxification. These promoters are heme oxygenase 1 (HO-1), glutathione s-transferase (GST), and NADPH quinine oxidoreductase 1 (NQO1) (Figure 4) [131]. Thus, the Nrf2 pathway is an important aspect of the cellular defense mechanism against oxidative stress [132].

### 3.1. Endogenous Antioxidants

The inherent antioxidative protective mechanism is composed of antioxidant enzymes such as superoxide dismutase (SOD), catalases (CAT), and glutathione peroxidases (GPx-1) [130]. In addition, low-molecular-weight nonenzymatic antioxidants include thiol antioxidants (Glutathione, α-lipoic acid), uric acid, and coenzyme Q10 (CoQ10) [133]. By scavenging excess ROS and limiting further generation of free radical species, these antioxidants collectively can prevent the detrimental effects of oxidative stress [134]. Antioxidants can also neutralize any free radical or a reactive species that can produce new free radicals [135].

#### 3.1.1. Antioxidant Enzymes

Superoxide Dismutase (SOD): SOD is a heterogeneous metalloprotein enzyme having four different types of metals at the center, i.e., Cu, Zn, Fe, Mg, and Ni with a crystalline nature. In the presence of these metal ion cofactors, SOD located in the cytosol and mitochondria catalytically converts O_2_^•−^ into O_2_ and H_2_O_2_ [136]. O_2_^•−^ is detoxified to yield H_2_O_2_ by Mn-SOD in the mitochondrial matrix or by Cu/Zn-SOD in the cytosol and intermembrane space, and H_2_O_2_ can also be transformed to ^•^OH in the presence of reduced transition metals [40]. Cu/Zn-SOD enzymes play a critical function in the first line of antioxidant defense [135].

Catalase (CAT): It is a tetrameric porphyrin-containing enzyme found mostly in the peroxisome that protects cells by converting H_2_O_2_ into H_2_O and O_2_ using either a Fe or Mn cofactor [137]. This mechanism prevents the formation of H_2_O_2_ and lowers the level of ROS; both are important mechanisms in the development of oxidative stress tolerance [135].

Glutathione Peroxidases (GPx): GPx is another intracellular enzyme that reduces ROS levels by conversion of H_2_O_2_ into H_2_O while oxidizing glutathione (GSH) to produce H_2_O and glutathione disulfide (GSSG) [138]. Several isoforms of GPx contain either five selenium cofactors or three noncysteine residues, which is important for enzyme activity. Most of these enzymes are found in the mitochondrial matrix, with a little quantity in the cytoplasm [139].

#### 3.1.2. Antioxidant Non-Enzymes

Glutathione (GSH): GSH is a tripeptide composed of amino acids, i.e., glycine, cysteine, and glutamic acid, and is the most abundant endogenous water-soluble antioxidant. GSH can directly neutralize ROS and is an important factor in the xenobiotic metabolism [140]. To maintain an intracellular reducing environment and counteract excessive generation of ROS, GSH works with three groups of detoxification enzymes. These enzymes include glutathione peroxidase (GPx), glutathione reductase (GR), and glutathione oxidase [139].

α-Lipoic Acid (ALA): ALA is categorized as sulfur-containing molecules that catalyze the oxidative decarboxylation of α-keto acids, such as pyruvate and α-ketoglutarate. As a universal antioxidant, oxidized lipoic acid and its reduced counterpart, dihydrolipoic acid (DHLA), can quench free radicals in both lipid and aqueous environments [141].

Uric Acid: Uric acid is a hydrophilic antioxidant produced during purine nucleotide metabolism that accounts for about 60% of the total blood serum-free radical scavenging activity. Uric acid is an effective electron donor and scavenger of a variety of ROS, including ^•^OH, O_2_^•−^, OONO^−^, HClO, and lipid peroxides. Complete scavenging of such species requires the participation of ascorbic acid and thiols in its cycle [142].

Coenzyme Q 10 (CoQ10): Coenzyme Q10 (CoQ10) or ubiquinol is another antioxidant enzyme cofactor involved in the mitochondrial ETC, which transfers electrons in complex I and complex II to complex III. CoQ10 is a lipid-soluble antioxidant present in all the cell membranes and inhibits lipid peroxidation [143]. In addition, other antioxidants, such as Vitamin E and C, require CoQ10 for their recycling and regeneration [144].

### 3.2. Exogenous Antioxidants

Dietary sources contain complex systems of multiple antioxidants that include vitamins (C, E, and A); carotenoids; and various polyphenols that the human body cannot synthesize. These antioxidants inhibit the initiation of the chain reactions or break the chain reactions by donating an electron to radicals, resulting in nonharmful species [4]. Furthermore, these exogenous antioxidants aid in the reinforcement and replenishment of the endogenous antioxidant, allowing the elimination of excess ROS/RNS [145].

Vitamins: Vitamin C (ascorbic acid) represents an efficient electron donor, converting free radicals to stable entities in the aqueous phase of the cytoplasm [146]. Tocopherols and tocotrienols are lipid-soluble forms of vitamin E, protecting the membrane lipids by inhibiting lipid peroxidation caused by oxidative and inflammatory reactions [147]. Vitamin A designates a family of unsaturated lipid-soluble organic compounds that include retinol, retinal, retinoic acid, retinyl palmitate, and many provitamin-A carotenoids, such as β-carotene [148]. Vitamin supplements are commonly used with an anticipation that they will protect cells and tissue from oxidative stress [149,150,151].

Carotenoids: Carotenoids are fat-soluble terpenoids containing conjugated trans double bonds. Carotenes (lycopene, β-carotene, or α-carotene) and xanthophylls (lutein, astaxanthin, fucoxanthin, capsanthin, zeaxanthin, and canthaxanthin) belong to the carotenoid family, widely present in red, orange, and yellow pigments in carrots; sweet potatoes; papaya; mangos; tomatoes; and oranges [152]. Carotenoids, acting as free radical scavengers and singlet oxygen quenchers, play a key role in inhibiting the oxidation of lipids [135]. In addition, these carotenoids inhibit apoptosis by preventing oxidative stress and display antioxidant and neuroprotective roles [153,154,155].

Polyphenols: Polyphenolic compounds are present in various fruits; vegetables; and beverages, such as grape juice, green tea, or coffee, and possess antioxidative, anti-inflammatory, and neuroprotective properties by scavenging free radicals [156]. Polyphenols have a wide range of aromatic structures, but the basic monomer in polyphenols is the phenolic ring. Depending on the strength of the phenolic ring into phenolic acids, they can be classified into phenolic acids, flavonoids, stiblins, phenolic alcohols, and lignans [157]. These polyphenols commonly include anthocyanins from berries, resveratrol found in grape skin or seeds, catechins from green tea, and curcumin isolated from the rhizome of the Indian spice turmeric Curcuma longa Linn [158]. Other most studied polyphenolic chemicals include chalcones, epigallocatechin gallates (EGCG), and quercetin [159]. These antioxidants detoxify various free radicals by scavenging or trapping them and by upregulating the activities of endogenous antioxidants [160]. These natural polyphenols also prevent oxidation of proteins, LPO, and show neuroprotective and neuroregenerative effects [133].

### 3.3. Synthetic Antioxidants

Synthetic antioxidants, modifications of natural antioxidants, or conjugates with other effective molecules have been prepared for better scavenging activity, bioavailability, and metabolic stability than natural antioxidants [161]. Synthetic antioxidants such as butylated hydroxyanisole (BHA), butylated hydroxytoluene (BHT), propyl gallate (PG), and tert-butyl hydroquinone (TBHQ) are widely used in the food industry to prevent lipid oxidation [162].

Recent research on synthetic antioxidant derivatives provides promising data against oxidative stress and multiple targets in neurodegenerative diseases. For example, synthetic compound 4-((5-(Tert-butyl)-3-chloro-2-hydroxy benzyl) amino)-2-hydroxybenzoic acid [163] and 1,3,4 oxadiazole compound A3 [164] showed significant antioxidative and neuroprotective effects. Synthesized docosahexaenoic acid (DHA)-acylated astaxanthin diesters (AST-DHA) showed substantially better effects than astaxanthin in reducing oxidative stress tau protein, enhanced learning and memory [165], and suppressing apoptosis of the dopaminergic neurons [166]. Similarly, the synthetic pyrazole derivative of curcumin (CNB-001) was demonstrated to suppress RNS generation with anti-inflammatory effect [167]. Synthetic derivatives of a natural phenolic compound such as caffeic acid phenethyl ester (CAPE) [168] or coumarin [169] also demonstrated to protect dopaminergic neurons by inhibiting p38 phosphorylation, increasing cell viability, and promoting antioxidant response.

Combination of novel synthetic pyrazole-containing compound 5-amino-1- phenyl-1H-pyrazole-4-carbonitrile (APPC) with lipoic acid, i.e., UPEI-800, showed synergistic neuroprotection both an in vitro hypoxia model and in vivo stroke model by reducing infarct volume [170]. A synthetic hybrid of antioxidants, i.e., coumarin and licochalcone A (Lico A), i.e., LM-031, has shown to inhibit Aβ aggregation in Aβ-GFP SH-SY5Y cells, scavenge ROS, promote neurite outgrowth, and activate the Nrf2-related antioxidant and antiapoptotic pathways [171]. Another hybrid compound (Dlx-23) developed by conjugating ALA and 3-n-butylphthalide (NBP), was shown to protect neuronal cell death, restore redox homeostasis, and synergistically prevent mitochondrial damage in a stroke model [172].

Synthetic nitrones are effective inhibitors of short-lived free radicals [173]. Due to their ability to react with free radicals to form a persistent nitroxide spin adduct; they can be used as an analytical tool for the detection and characterization of free radicals using Electron Paramagnetic Resonance (EPR) spectroscopy [174]. Synthetic nitrone derivatives showed antioxidative and neuroprotective effects in various neurodegenerative disease conditions [175,176].

Synthetic edaravone scavenges free ^•^OH radicals and OONO^−^ radicals, which are highly associated with neuronal damage/death in cerebrovascular disorders such as ischemic strokes and degenerative neurological disorders such as ALS [177]. It exerts neuroprotective and antioxidant effects and delays disease progression by limiting the extent of lipid peroxidation and cell membrane damage from oxidative stress [178].

In recent years, mitochondrial-targeted antioxidants have been successfully developed. For e.g., synthetic analogs of CoQ10, idebenone, and mitoquinone (MitoQ) demonstrated effective amelioration of mitochondrial ROS [179], DNA damage, neuroinflammation, and prevented neuronal degradation [180]. Idebenone is characterized by a shorter and less lipophilic tail than CoQ10, and MitoQ is composed of ubiquinone and triphenylphosphonium (TPP+) [181]. Plastoquinone derivatives, i.e., SkQ1 and SkQR1 molecules that contain an antioxidant moiety linked to a lipophilic cation, also demonstrated a neuroprotective effect [182]. Synthetic arylidenmalonate derivative 5-(3,4-dihydroxybenzylidene)-2,2-dimethyl-1,3-dioxane-4,6-dione (KM-34) also showed significant antioxidant property, mitoprotection and neuroprotection in vitro and in vivo models [183]. 

## 4. Preclinical Studies with Antioxidant Agents

### 4.1. Antioxidant-Based Therapy in Neurodegenerative Diseases

With a strong rationale that oxidative stress is a key component of neurodegenerative diseases, antioxidants of different types, either alone or in combination, natural and synthetic have been tested in neurodegenerative disease models. In general, in AD models, the treatment with antioxidants produced favorable outcomes. For e.g., the treatment with CoQ10 or lipoic acid increased the levels of ATP and SOD and reduced the levels of Apolipoprotein E (ApoE) and Aβ fragments [184]. The treatments also reduced the levels of phosphorylated tau and neuroinflammatory factors [185] and improved hippocampal synaptic plasticity [186]. Similarly, the treatment with carotenoids inhibited the markers of oxidative stress [137,187] and the AD marker proteins, improved memory loss, and reduced inflammation [188,189,190]. Polyphenols such as resveratrol [191], curcumin [192,193], and anthocyanin [121] have been shown to attenuate glutamate-induced excitotoxicity, increased antioxidant capacity and mitophagy [194], and rescue cell death in AD models [195,196,197]. The nutritious mushroom, *hericium erinaceus* is a source of exogenous antioxidants and has been shown to possess neuroprotective and anti-inflammatory properties [198]. In a sporadic AD model, *hericium erinaceus* treatment reduced behavioral abnormalities, hippocampus neuronal degeneration, and AD markers [199]. A few combination therapies such as ubiquinol and ascorbic acid [200], lycopene with vitamin E [201], CoQ10 and Omega-3 [202], and resveratrol and curcumin [203] reported to having a synergistic beneficial effect on reducing amyloid plaques and tau hyperphosphorylation in transgenic or sporadic models of AD. A synthetic derivative of CAPE termed FA-97 was developed by Wan et al. and has been shown to attenuate H_2_O_2_-induced apoptosis and suppress the levels of ROS, MDA, and protein carbonyl; and induce the cellular antioxidant levels in an in vitro study [204].

In PD models, supplements of vitamins E [205] and C [206,207] and CoQ10 [208] have been shown to restore corticostriatal synaptic plasticity, reduce dopaminergic cell death in the substantia nigra, microglial activation and astrogliosis, and improve behavioral parameters. ALA was shown to suppress oxidative stress, mitochondrial dysfunction, and glutamate-induced toxicity [209,210]. The treatment with other antioxidants, crocin [211] or fucoxanthin [212], was also shown to suppress autophagy and improve behavioral alterations, homeostasis, and mitochondrial enzyme function. In the pesticide-induced PD model, treatment with resveratrol [213] improved lifespan and behavioral deficits [214] via Nrf2 activation [215]. Further, the combinatorial treatment of quercetin and piperine (bioavailability enhancer) significantly improved behavioral abnormalities [216]. A recent study has reported that the treatments with synthetic chalcone derivate and 2-Hydroxy-40-methoxychalcone (AN07) reduced ROS level, stimulated Nrf2 pathways, increased GSH levels, and decreased inflammatory factors, thus favoring recovery [217]. In dopaminergic catecholaminergic (CATH.a) cells, a novel synthetic morpholine-containing chalcone (KMS99220) was shown to reduce oxidative stress effectively and protein aggregation, potentiate the Nrf2 mechanism and lower intracellular aggregation of α-synuclein [218]. In another example, Drummond et al. reported the antioxidant ability of a novel synthetic flavonoid, Proxison (7-decyl-3-hydroxy-2-(3,4,5-trihydroxyphenyl)-4-chromenone), and demonstrated enhanced cellular uptake, radical scavenging capabilities and neuroprotection against cell loss in a zebrafish model of dopaminergic neurodegeneration [219].

In ALS, mutation of the SOD1 genes reduces the antioxidant enzyme activity and hence is ineffective in lowering the ROS levels [220]. Curcumin has been shown to inhibit aggregation and fibrillation of SOD1 amyloid fibrils, lowering amyloidogenicity and neurotoxicity [221]. In a mouse model of ALS, treatment with anthocyanin-enriched extracts from strawberries was found to delay the disease onset, improve grip strength, reduce spinal motor neuron death, and preserve neuromuscular junctions (NMJs) [222]. Zhao et al. discovered that EGCG treatment stabilizes SOD1 conformation against misfolding and inhibits apo-SOD1 aggregation [223]. EGCG was found to have a substantial binding affinity for mutant SOD1, which reduces its toxic aggregate formation [224]. Phenolic compounds, quercitrin, quercetin 3-β-d-glucoside, and EGCG have been found to inhibit H_2_O_2_-induced misfolding and aggregation of A4V SOD1 [225]. Kaempferide and kaempferol are active ingredients of Brazilian green propolis that possess antioxidative properties and were shown to prevent SOD1 intracellular aggregates in a mutant SOD1-induced N2A cellular model [226]. Other studies also reported that the treatment with antioxidants (e.g., fisetin or protocatechuic acid) improves survival rate, attenuates motor impairment, reduces astrogliosis and microgliosis in the spinal cord, protects the spinal motor neurons from apoptosis, and regulates redox homeostasis by lowering the levels of both mutant and wild-type human SOD1 [227,228].

### 4.2. Antioxidant-Based Therapy in Neurological Injury

This review selected stroke, SCI, and PNI as examples where oxidative stress plays a key role in the early pathological and progressive degeneration following the acute event; this mechanism is also relevant to the acute TBI [123]. Treatments with several types of antioxidants, including α-lipoic acid (ALA) [229], α-tocopherol [230], vitamin C [231], crocin [232], resveratrol [233], and (−)-Epicatechin [144], have been shown to significantly reduce infarct volume, brain edema, oxidative damage, and apoptosis. In addition, the treatments protected the BBB integrity and promoted neurological recovery in stroke model studies. In other studies, pretreatment with natural free radical scavenger (e.g., ginkgo biloba extracts (Egb-761) [234] and astaxanthin [235]) has been shown to significantly ameliorate ischemic injury and reduce infarct volumes and brain edema, accompanied by alleviated oxidative stress, and upregulation of expression of brain-derived neurotrophic factor (BDNF) and nerve growth factor (NGF) mRNA.

In several studies, the efficacy of antioxidant treatment has been examined in animal models of acute SCI. They have been found to inhibit the expression of proapoptotic proteins (Bax and Caspase-3), increase the level of antiapoptotic protein (Bcl-2), reduce the level of MDA, and improve the activities of SOD and GSH (e.g., CoQ10) [236]. For example, the rats with SCI treated with vitamin E-enriched diet showed accelerated bladder recovery and improved locomotor function [237]. Treatment either with β-carotene or lycopene was also shown to reduce oxidative damage, mitochondrial dysfunction, cell apoptosis, and hind limb motor disturbances [238]. The treatment also inhibited inflammation by blocking the nuclear factor kappa B (NF-κB) pathway [239]. Antioxidants such as curcumin derivative, EGCG, or astaxanthin have been shown to reduce inflammation [240], promote regeneration, provide neuroprotection, and ultimately improve functional recovery [241,242]. Similarly, the treatment with resveratrol was shown to reduce the levels of inflammatory cytokines and inhibit cell death [243], improve motor function [244] via activation of the Sirtuin 1 (SIRT-1)/NF-κB signaling pathway [245], Beclin-1 and LC3-B, key proteins of autophagy [246], or the SIRT1/Adenosine 5′ monophosphate-activated protein kinase (AMPK) signaling pathway [247]. Quercetin treatment in SCI models was shown to reduce necroptosis of oligodendrocytes, which prevented axonal loss [248] and also suppressed macrophages/microglia polarization to proinflammatory M1 phenotype [249]. The combination treatment with ascorbic acid and taurine (nonproteogenic essential amino acid) showed synergistic protection against apoptotic, inflammatory, and oxidative stress markers in SCI-induced rats [250]. In PNI, compounds having antioxidative properties such as vitamins, carotenoids, enzymes, and proteins have been demonstrated to facilitate the process of nerve repair [127].

### 4.3. Clinical Trials with Antioxidants

Promising data from preclinical studies led clinical trials to determine the efficacy of antioxidants in different neurodegenerative diseases/injuries, primarily with few commonly used antioxidants such as curcumin, vitamin E, lipoic acid, and CoQ (Table 1 and Table 2).

A phase II clinical trial study with oral dosing of curcumin in AD patients was shown to reduce cognitive deterioration but did not improve cognition [251]. Curcumin oral supplementation also demonstrated a slight slowdown in the disease progression in ALS patients [252]. Treatments with resveratrol [253,254] and EGCG [255] have been shown to attenuate Aβ_1–40_ and slow cognitive decline in AD patients, and in stroke patients, reduce the levels of matrix metalloproteinase-9 (MMP-9) and matrix metalloproteinase-2 (MMP-2). However, intranasal administration of GSH did not show the effect of the treatment on motor scores in PD patients [256,257]. High-dose treatment of CoQ10 in the idiopathic PD participants showed improved unified PD rating scale (UPDRS); however, it was indicated that the high-dose of CoQ10 (2400 mg/day) could increase the risk of oxidative damage in the long run [258]. In another clinical trial with CoQ10, although its dose was found safe and well-tolerated, it did not show any therapeutic benefits; hence, the study was terminated [259]. Similarly, a high dose of CoQ10 treatment showed a decrease in the ALS Functional Rating Scale-revised (ALSFRSr) score; however, the subsequent analyses revealed no significant differences compared to the placebo control [260]. Ginkgo biloba treatment in older patients with cognitive impairment also did not improve cognition compared to placebo [261]. Clinical trial on edaravone demonstrated a significant reduction in the ALSFRSr score in ALS patients compared to placebo group [262,263]. Furthermore, edaravone has been shown effective in recovery in stroke patients and reduce the MMP-9 levels [264,265]. In addition, the combination treatment, edaravone with (+)- borneol, a food additive, has been proven to be safe and well-tolerated in stroke patients [266] and is currently under a phase II clinical trial in patients suffering from intracerebral hemorrhage [267].

The combination treatments such as lipoic acid and omega-3 fatty acids, i.e., fish oil, given to AD patients were found to slow the cognitive and functional decline as compared to the placebo [268]. Similarly, vitamin E, the AD drug memantine (brand name: Namenda), or their combination was shown to slow down the clinical progression of AD. Interestingly vitamin E treatment resulted in a slower functional deterioration than the combination [269].

A combination treatment consisting of vitamin E, vitamin C, ALA, and CoQ did not show the effect on amyloid or tau pathology biomarkers in the cerebrospinal fluid (CSF). Furthermore, the treatment with CoQ did not improve oxidative stress or neurodegenerative indicators [270]. On the other hand, omega-3 fatty acids and vitamin E co-supplementation in PD patients resulted in a significant improvement in the UPDRS and favorable effect on total antioxidant capacity (TAC) and GSH levels compared to placebo but did not affect the oxidative stress indices or lipid profiles and inflammatory factors [271]. Daily treatment of the combination of ginkgo biloba (Egb761) and aspirin in stroke patients alleviated cognitive and neurological impairment after acute ischemic stroke without increasing the risk of vascular injury [276]. In SCI patients, dietary supplementation containing curcumin and omega-3, vegetation protein powder, antioxidant network capsule, and chlorella tablet were reported to reduce inflammatory mediators and improve depressive behavior [277]. Overall, the clinical trial results showed some trend towards a positive outcome, particularly the changes in the pathological markers and a few studies, improvements in functional outcome, thus indicating the potential of antioxidants to mitigate oxidative stress in humans. However, the results also highlighted the need to improve their therapeutic efficacy and make the clinical outcomes conclusive and reproducible, and importantly achieve functional recovery. To that end, in addition to developing more potent and target-specific antioxidants, drug delivery approaches have also been explored.

### 4.4. Drug Delivery Challenges

Despite promising results in preclinical models of neurodegenerative diseases, not all the clinical trial results were definitive. In general, antioxidant compounds were found to reduce clinical signs and symptoms only [252] but unable to stop the disease progression or reverse it [261]. Vitamins and flavonoids are still used but mostly as dietary supplements, which may act as prophylactic with long-term use. Edaravone (free radical scavenger) is the only Food and Drug Administration (FDA)-approved antioxidant treatment, and it is used to help people recover from stroke in Japan and is used to treat the early stages of ALS in the US and Japan, but it does not affect the disease progression in late-stage ALS, thus benefiting only 5% of ALS patients [278]. In Europe, the use of Radicava medication (active substance of edaravone) has been withdrawn from the marketing authorization, since the data did not show a positive benefit-risk balance [279]. There are various challenges associated with effective drug delivery of antioxidants that may be impeding their clinical translation.

- Low permeability to the CNS: The presence of a physiological barrier such as the BBB or spinal–blood barrier (SBB) restricts the accessibility of antioxidant compounds to the CNS and hence could not achieve a prolonged therapeutic dose to impart an antioxidant effect in chronic neurodegenerative diseases [280]. In certain pathological conditions, the BBB/SBB may be compromised due to inflammation or injury (e.g., stroke and spinal cord injury) but still may not be able to achieve the desired dose for a prolonged period due to transient and limited permeability of the BBB/SBB, giving a narrow time window for delivery of therapeutics [281].
- Low bioavailability: Most antioxidants are given orally, and they are insoluble or unstable in a gastric environment that could result in low bioavailability to provide high systemic levels for transport to the CNS at effective doses [282,283]. Antioxidant compounds that are administered via systemic routes have short half-lives [284], which could also limit their transport to the CNS.
- Low catalytic activity: High doses of antioxidant compounds are needed to detoxify the effect of free radicals, which could not be given to humans because of the dose-limiting toxicity [285]. Noncatalytic antioxidant becomes ineffective, once these molecules interact with free radicals [286], and hence, maintaining high antioxidant levels in the target tissue to counteract free radicals that are formed over a period of time in chronic conditions could be challenging.
- Toxicity: Due to toxicity concerns, human doses could have been significantly lower than those used in animal model studies. This could also constrain the duration of treatment necessary to see the beneficial outcome in clinical trials [287].
- Oxidative stress target and other factors: Although oxidative stress is considered as the driving force behind neurodegenerative diseases, there could be other cofounding pathological factors in humans that may not have been targeted solely by antioxidants [288,289]. In addition, the question raised is also how close animal models are to human pathology [290].

## 5. Antioxidant-Based Nanotherapy

**Figure 5:**
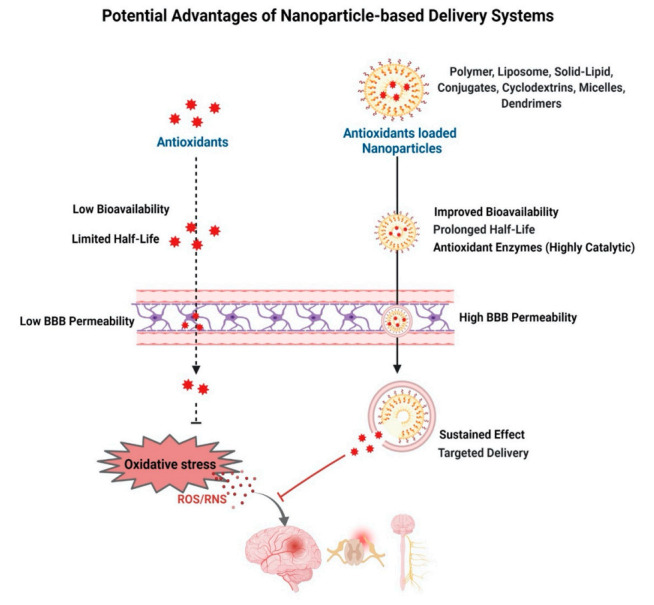
Antioxidant-based nanotherapy. Schematic depicting advantages of delivery of antioxidant-loaded nanoparticles to improve half-life of antioxidants and their ability to cross the BBB, improve bioavailability, and sustain the effect, thus effectively neutralizing oxidative stress in neurodegenerative diseases.

To overcome the limitations of natural and synthetic antioxidants, significant efforts have been made to improve their efficacy using drug delivery approaches. These include exploring nanocarriers of different polymeric materials, conjugates, and complexes [291,292] to improve their stability, half-lives, transport to the CNS, and sustained their effect in the target tissue (Figure 5).

Due to its broad pharmacological effects, including anti-inflammatory and antioxidant properties, curcumin has been widely investigated in clinical studies. To overcome its low water solubility, poor bioavailability, and rapid metabolism, curcumin is formulated as nanocurcumin using different nanocarriers, such as liposomes, polymers, conjugates, cyclodextrins, micelles, dendrimers, and nanoparticles [293]. Transferrin-conjugated poly (lactic *co*-glycolic acid) (PLGA) nanoparticles have been demonstrated to improve the bioavailability of curcumin to the brain and reduce Aβ deposition and tau hyperphosphorylation in the AD model [294]. Similarly, different formulations of nanoparticles have been shown to inhibit aggregation of Aβ and reduce depressive-like behavior and oxidative stress in AD models [295,296]. Intra-arterial administration of resveratrol (RES)-encapsulated nanoparticle (RES-NP) in a rat transient middle cerebral artery occlusion (t-MCAO) enhanced the resveratrol bioavailability and its brain-penetration, resulting in reduced infarct volume, and attenuated oxidative stress [297], brain edema, and neuronal apoptosis. The treatment also contributed to neurogenesis, leading to improved neurological recovery [298]. In a cerebral palsy rabbit model, intravenous treatment of dendrimer-based N-acetyl-l-cysteine (NAC) [299], a glutathione precursor with antioxidant and anti-inflammatory properties [300], reduced neuroinflammation and neurological injury, and improved motor function. In general, formulating antioxidants in nanocarriers has enhanced their efficacy due to better stability and/or improved transport to the CNS than free antioxidants [301,302,303,304,305,306,307,308,309,310,311,312,313,314,315,316,317,318,319,320,321,322,323,324,325,326,327]. Nanocurcumin has been evaluated as an add-on therapy to Riluzole in a pilot randomized clinical trial for safety and efficacy in ALS [272] and AD patients as dietary supplements [274]. In another study, solid–lipid curcumin showed significantly improved cognition and mood in healthy older population [273].

Edaravone-loaded ceria nanoparticles have demonstrated to cross the BBB via receptor-mediated transcytosis and protect the BBB [328]. In addition to the antioxidant property of ceria nanoparticles, edaravone provided its effect against oxidative stress in a stroke model [328]. Jin et al. demonstrated that the treatment with edaravone-encapsulated agonistic micelles caused rapid infarct volume reduction, prolonged survival, improved axonal remodeling, and reduced behavioral deficits than free edaravone-treated animals [329]. Wang et al. reviewed nanotechnology-based strategies for the treatment of ALS, including antioxidant agents [330]. Nanoparticle-loaded edaravone has been tested on the postoperative effects in patients with cerebral hemorrhage. The nanoparticle-loaded edaravone showed reduced edema as compared to free edaravone treated group, significantly improved neurological function, and reduced the production and release of interleukin and tumor necrosis factor, which was considered beneficial to protect healthy brain tissue and other organs, and conducive to the recovery and healing [275].

### Antioxidant Enzymes

When they interact with free radicals, natural or synthetic antioxidants become inactive [331]. To continue to neutralize free radicals formed over a prolonged period, in chronic disease conditions, therapeutic levels of these antioxidants need to be maintained, which could be challenging, as repeated and high dosing cause dose-limiting toxicity in humans [331]. The main advantage of antioxidant enzymes is their catalytic mode of action [6]; hence, they can effectively neutralize free radicals at low doses. However, due to their short half-lives (5–11 min) [332], exogenously delivered antioxidant enzymes are ineffective in combating oxidative stress. Modifications such as PEGlylation and lecithinization improve their stability in the circulation [333] and fusion with cell membrane-penetrating peptides like a transactivator of transcription peptide or tetanus toxin fragment increases their ability to cross the BBB [334]. However, there are limitations to these modifications. Although PEGylated SOD (PEG-SOD) increases the enzyme’s stability in the circulation from 6 min to 36 h, PEG limits the permeability of the conjugated SOD across cerebral cell membranes [335]. Similarly, a chemical reaction involved in the fusion of different cell-penetrating or cell-specific peptides could cause denaturation and loss of enzyme activity [336]. In addition, the newly formulated hybrid enzyme could trigger immune-mediated anaphylactic responses to patients [337]. Intravenous delivery of SOD loaded into liposomes has shown to partially inhibit the infarct volume, but instability of liposomes in vivo (half-life ~4.2 h) limits the duration of SOD activity and, hence, its efficacy [338,339].

The recent effort includes formulations of antioxidant enzymes, SOD1, and catalase by electrostatic coupling of enzymes with cationic block copolymers called nanozymes [340]. In mice, this formulation demonstrated increased stability of enzymes in both blood and the brain and showed increased accumulation in the brain tissues than enzyme alone treated animals [340]. In a rat MCAO model, nanozymes reduced I/R-induced tissue injury and improved the sensorimotor functions [341]. In a moderate SCI rat model, treatment with nanozymes showed a recovery of locomotor functions, reduction of swelling, and post-traumatic cysts in the spinal cords of the treated animals [342]. Muzykantov’s group reviewed different nanocarriers to deliver antioxidant enzymes for vascular targeting in oxidative stress conditions associated with cardiovascular, pulmonary, and nervous systems [343].

Our research group has been investigating the efficacy of antioxidant enzymes encapsulated in PLGA-based sustained release nanoparticles. The neuroprotective efficacy of the SOD-encapsulated nanoparticles (nano-SOD) was initially demonstrated in the H_2_O_2_-induced oxidative stress model in human neuronal cells and, subsequently, with the CAT-encapsulated nanoparticles (nano-CAT) in human astrocytes [344,345]. In the MCAO model in rats, intracarotid administration of nano-SOD following 1 h of ischemia inhibited reperfusion injury. The treatment demonstrated improved neurological recovery and survival compared to controls (saline or SOD solution). There was evidence of neuronal recovery and regeneration with time in the above study [346]. The follow-up study in a thromboembolic rat stroke model, where tissue plasminogen activator (t-PA) was administered first for clot lysis followed by nano-SOD/CAT, both via the carotid artery, demonstrated the protective effect of the treatment. Significantly, the t-PA + nano-SOD combination treatment stimulated the migration of stem/progenitor cells from the subventricular zone and circulation, promoting neurogenesis. In contrast, this process was inhibited in the animals which received t-PA only treatment [347]. The above sequential treatment also inhibited edema formation, suggesting protection of the BBB from reperfusion injury [347]. In a separate study, we demonstrated aggravation of the BBB permeability when t-PA alone was administered via the carotid artery in the same thromboembolic rat stroke model [348]. Thus, the delivery of antioxidant enzyme nanoparticles in the above sequential treatment study protected the BBB from reperfusion injury and, also, from the effect of t-PA.

**Figure 6:**
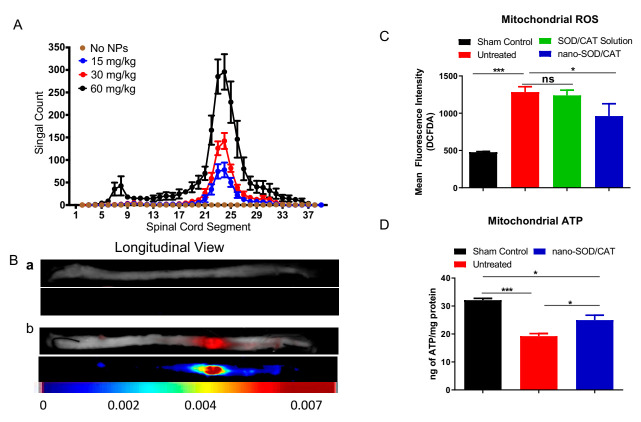
Antioxidant enzyme-based nanotherapy for spinal cord injury: Localization of nanoparticles at the lesion site following intravenous administration. Nanoparticles were injected 6 h post-injury, and spinal cords were analyzed for localization of the nanoparticles. Nanoparticles contained a near-infrared dye, and the spinal cords were analyzed 24 h after the injury using the Maestro Optical Imaging System site. Reproduced with permission from [350], copyright 2019 Elsevier. (**A**) Dose-dependent localization of nanoparticles at the lesion site. (**B**) Images of the spinal cord taken with Maestro Optical Imaging (**Ba**) Normal spinal cord without injury and nanoparticles. (**Bb**) Injured spinal cord from the animals that received dye-loaded nanoparticles intravenously. Efficacy of nano-SOD/CAT treatment (**C**)-treated animals show reduced mitochondrial ROS levels. (**D**) Mitochondrial isolated from the spinal cord of the treated animals show more ATP production capacity than those isolated from the spinal cords of untreated animals. * *p* < 0.05; *** *p* < 0.001 Reproduced with permission from [350], copyright 2019 Elsevier.

In our recent study, we demonstrated that intravenous administration of nano-SOD/CAT, 6-hr following injury in a rat severe contusion model of SCI, partially attenuated mitochondrial dysfunction, reduced ROS levels, and the expression of apoptotic factors (Figure 6C). Further, the isolated mitochondria from the spinal cords of the treated animals demonstrated reduced ROS activity and higher ATP production capacity than those isolated from untreated control animals (Figure 6D). The overall effect of the treatment was found to be the protection of the injured spinal cord from cell apoptosis and further degeneration [349]. The protective effect of the treatment was seen across the entire spinal cord as there was reduced expression of apoptotic factors, suggesting that the treatment significantly attenuated the progression of secondary injury. Neuroprotective efficacy of the nano-SOD/CAT in the above SCI model study is attributed to the protection of the encapsulated enzymes and sustained antioxidant effect at the lesion site [350] (Figure 6). Since nano-SOD/CAT is formulated with PLGA, an FDA-approved polymer, its translational potential is high.

## 6. Concluding Remarks/Future Perspective

Effective treatment for neurodegenerative diseases is a clinically unmet need. Substantial evidence supports the hypothesis that oxidative stress plays a key role in disease progression; hence, antioxidant treatment could provide a potential solution. However, several challenges, including inadequate dosing, low bioavailability, limited transport to the CNS and transient retention, and low antioxidant capacity to completely detoxify the effect of free radicals, could have limited their translation to clinical practice. In this regard, nanoparticle-based drug delivery systems could address some of the above issues. Antioxidant enzymes hold promise due to their high catalytic activities; therefore, much work has been done in recent years to develop the nanotherapy-based approach to delivering these antioxidant enzymes. Since oxidative stress is a common pathophysiological process in multiple diseases, an effective antioxidant system could have broad therapeutic applicability in many clinical settings.

## Figures and Tables

**Table 1:** Clinical trials of antioxidants in neurodegenerative diseases.

Antioxidants	Route	Disease Patients	Dosage	Follow Up Period	No. of Patients	Outcome	References
Curcumin	Oral	AD ALS	1.5 g/d 100 mg/d	6 months 9 months	34 42	Reduced cognitive deterioration Slowdown in disease progression	[251,252]
Resveratrol	Oral	AD	1 g/d	52 weeks	119	Decreased Aβ_1–40_ and MMP-9 levels in CSF Slowed cognitive decline	[253]
GSH	Intranasal	PD	300 mg/d or 600 mg/d thrice 100 mg/d or 200 mg/d thrice	3 months	30 45	Safety and tolerability No significant differences between groups No effect on motor function	[256,257]
CoQ10	Oral	PD PD ALS	400, 800, 1200, and 2400 mg/d 1200 mg/d or 2400 mg/d 1800 mg/d and 2700 mg/d	10 weeks 16 months 9 months	16 600 105	Improved UPDRS, Reduced F2-isoprostanes No therapeutic benefit Decreased ALSFRSr No significant differences between groups at high dose	[258,259,260]
Ginkgo biloba	Oral	AD	120 mg/d twice	8 years	3069	No improvement in cognition	[261]
Edaravone (FDA Approved in 2017)	Intravenous	ALS	60 mg/d	24 Weeks	137	Decreased ALSFRSr	[262,263]
Lipoic acid and, Omega-3 fatty acids	Oral	AD	600 mg/d 675 mg docosahexaenoic acid (DHA) 975 mg eicosapentaenoic acid (EPA)	12 months	39	Slowed cognitive and functional decline	[268]
Vitamin E and, Memantine	Oral	AD	2000 IU/d20 mg/d	5 years	613	Slower functional deterioration in Vitamin E group	[269]
Vitamin E, Vitamin C, ALA, and CoQ	Oral	AD	800 IU/d 500 mg/d 900 mg/d 400 mg/d thrice	16 weeks	78	No effect on amyloid or tau pathology biomarkers	[270]
Omega-3 fatty acids and, Vitamin E	Oral	PD	1000 mg 400 IU	12 weeks	60	Improved UPDRS, TAC and GSH	[271]
Nanocurcumin and, Riluzole	Oral	ALS	80 mg/d 50 mg/d twice	12 months	54	Safety and tolerability Increased survival probability of ALS patients	[272]
Curcumin Formulation (Longvida) Solid-Lipid Curcumin	Oral	AD Control	2000 mg–3000 mg/d 400 mg/d	9 months 4 weeks	26 60	Not provided Improved cognition and mood	[273,274]

**Table 2:** Clinical trials with antioxidants in neurological injury.

Antioxidants	Route	Disease Patients	Dosage	Follow Up Period	No. of Patients	Outcome	References
Resveratrol	Oral/ Infusion	Stroke	2.5 mg/kg	0–2 h of stroke onset	312	Decreased MMP-9 and MMP-2 levels	[253,254]
EGCG	Intravenous/ oral/ infusion	Stroke	500 mg	0–5 h of stroke onset	371	Decreased MMP-9 and MMP-2 levels	[255]
Edaravone	Intravenous	Stroke	30 mg 60 mg	6 months 12–24 h of stroke onset	40163	Effective recovery Decreased MMP-9 levels	[264,265]
Edaravone Dexborneol	Intravenous	Stroke Intracerebral Hemorrhage	12.5 mg, 37.5 mg or 62.5 mg every 12 h for 14 days 37.5 mg every 12 h for 14 days	3 months NA	385390 (estimated)	Safe and well tolerated No Recruitment	[266,267]
Nanoparticle-loaded Edaravone	Intravenous	Cerebral Hemorrhage	25 mg	3 weeks	120	Reduced edema Improved neurological function Reduced interleukin and tumor necrosis factor	[275]
Ginkgo biloba and, Aspirin	Oral	Stroke	450 mg 100 mg	6 months	348	Alleviated cognitive and neurological impairment	[276]
Omega-3 pill Vegetation Protein Powder InflanNox (curcumin) capsuleAnti-oxidant Network capsule Chlorella tablet	Oral	SCI	500 mg/d EPA, 250 mg/d DHA, thrice 45 g/d 400 mg/d thrice 615 mg/d twice 1000 mg/d, 6 times	3 months	20	Improvement in behavior Modification in neuroactive compounds Reduction in IL-1β	[277]

## Abbreviations

ALA	α-Lipoic acid
ALSFRSr	Amyotrophic Lateral Sclerosis Functional Rating Scale Revised
APP	Amyloid Precursor Protein
ARE	Antioxidant Response Element
BBB	Blood-Brain Barrier
CAT	Catalase
CNS	Central Nervous System
CoQ10	Coenzyme Q10
EGCG	Epigallocatechin Gallates
ETC	Electron Transport Chain
GSH	Glutathione
GST	Glutathione S-Transferase
GPx	Glutathione Peroxidases
GR	Glutathione Reductase
HO-1	Heme Oxygenase 1
4-HNE	4-Hydroxynonenal
KEAP1	Kelch-like ECH-Associated Protein 1
LDL	Low-Density Lipoproteins
LPO	Lipid Peroxidation
MCAO	Middle Cerebral Artery Occlusion
MDA	Malondialdehyde
MMP	Matrix Metalloproteinases
NF-κB	Nuclear Factor Kappa B
NFTs	Neurofibrillary Tangles
Nrf2	Nuclear Factor Erythroid 2-Related Factor 2
NQO1	NADPH Quinine Oxidoreductase 1
PLGA	Poly(Lactic-co-Glycolic Acid)
PNS	Peripheral Nervous System
RA	Retinoic Acid
ROS	Reactive Oxygen Species
RNS	Reactive Nitrogen Species
SIRT-1	Sirtuin 1
SOD	Superoxide Dismutase
TAC	Total Antioxidant Capacity
UPDRS	Unified Parkinson’s Disease Rating Scale
